# A Comparison of Total Antioxidant Capacities of Concord, Purple, Red, and Green Grapes Using the CUPRAC Assay

**DOI:** 10.3390/antiox2040257

**Published:** 2013-10-17

**Authors:** Connor M. Callaghan, Robert E. Leggett, Robert M. Levin

**Affiliations:** 1 Albany College of Pharmacy and Health Sciences, 106 New Scotland Ave, Albany, NY 12208, USA; E-Mail: connor.callaghan@acphs.edu; 2 Stratton VA Medical Center, 113 Holland Ave, Albany, NY 12208, USA; E-Mail: robert.leggett@va.gov

**Keywords:** grape, antioxidant, oxidative stress, CUPRAC assay, carbohydrates

## Abstract

Considering how popular grapes are in terms of their antioxidant benefits, we compared concord, purple, red, and green grapes for total antioxidant capacity (TAC) and carbohydrate concentration. All grapes were acquired from commercial sources and samples of each were separated into skinned and not skinned groups. Each whole grape and the skins were individually homogenized and then separated into pulp and supernatant fractions. Each fraction was analyzed for total TAC and carbohydrates. The concord grapes and purple grapes had significantly higher TAC in the homogenates than did the red or green grapes. The concord grapes and green grapes had significantly higher TAC in the pulp than in the cytosol whereas the red and purple grapes had approximately the same amount. The majority of the TAC of the purple and red grapes was in the skin whereas the concord and green grapes had approximately the same TAC in the skin and pulp. The concord and purple grapes had the highest TAC when compared to the red and green grapes, whereas the red and green grapes had approximately the same total TAC.

## 1. Introduction

Grapes are frequently touted as excellent sources of antioxidants [1,2,3] which are molecules involved in protecting cells against oxidative stress. Since oxidative stress leads to cell death and has been linked to a variety of diseases ranging from cancer and heart disease to Alzheimer’s disease and anxiety disorders [4,5,6,7,8,9], it cannot be overstated how important of a role antioxidants play in the body. Due to their ability to neutralize free radicals and stabilize oxidative stress [10], antioxidants have experienced a surge in popularity. Everything from dietary supplements to tea, wine, and various foods have been lauded for their antioxidant properties. In addition, it is clear that the natural antioxidant mechanisms of the body including superoxide dismutase and catalase decrease with age in both men and women making it even more important for persons to take antioxidant supplements as they age [11,12,13].

In the case of grapes, people tend to associate the differently colored grapes with varying levels of antioxidant capacity (TAC) with the rule of thumb being that the darker grapes have more TAC than the lighter ones. This general consensus is worth evaluating because the different colors may be more indicative of different classes of antioxidants rather than varying levels of antioxidants. It is known that the polyphenols in purple and red grapes consist predominantly of anthocyanins whereas in green grapes flavanols constitute the majority of the polyphenols [9,14,15].

In the current study, four different groups of grapes were analyzed by the CUPRAC biochemical assay for total TAC [16]: concord, purple, red, and green grapes. These are the four major types of table grapes which are grapes produced for the purpose of consumption as fresh grapes. Purple, red, and green grapes were of the seedless variety while the concord grapes had seeds. Even though the grapes are considered seedless, dissection reveals that there are several small, soft seeds in the red and green grapes. The softness of the seeds prevents them from being noticed during consumption. The interiors look virtually identical for these two grapes. The purple grapes, however, have much tinier seeds. The concord grapes have fairly large semi-hard seeds. The concord grapes were included because of the widespread belief that Concord grape juice has significant TAC and health benefits [17,18,19,20].

Due to the potential for antioxidants to protect against the onset of various diseases, it is important to better understand grapes as a vehicle for these antioxidants. The popularity of grapes makes it worthwhile to analyze and categorize the differences in antioxidants between the different colors of grapes in order to satiate the burgeoning demand of health awareness regarding common foods. Many different types of foods are commonly advertised as being rich in antioxidants, yet the amount of antioxidants is never explicitly stated. This fact might compel a curious person to wonder what the antioxidant activities are for the most common foods promoted for their antioxidants, such as grapes. It should also be noted that antioxidants have variable effectiveness *in vivo*, which is to say that total antioxidant capacity does not necessarily correlate directly with how beneficial different antioxidants may be in the human body [21,22].

## 2. Materials and Methods

All methods were approved by the Stratton VA Medical Center R&D Committee. Four different grapes (concord, purple, red, and green) were obtained from the supermarket for analysis using the CUPRAC biochemical assay for total TAC [22,23,24,25]. A carbohydrate analysis was also performed in order to normalize the antioxidant levels by carbohydrate [21,26]. Our use of carbohydrates to normalize the antioxidant capacity of the grapes gave us a quantitative basis for comparing these different grapes. Three different groups were created: one where the whole grapes were homogenized; the second where the skin was removed and the rest of the grape was homogenized; and the third where the second homogenate was centrifuged and the cytosol was analyzed. All grapes were homogenized at a concentration of 500 mg/mL in a 0.05 M Tris buffer (pH 7.6). The homogenates of the skinless grapes were centrifuged at 15,000× *g* for 20 min and separated into the pellet (pulp) and supernatant (cytosol). The skin was also homogenized and analyzed as stated above.

For the CUPRAC assay, all samples and standards were prepared in duplicates in which 150 μL of the sample or standard was mixed with 150 μL each of 1 M ammonium acetate, 7.5 mM neocuproine, and 10 mM copper (II) chloride dihydrate. The standard curve was established using 1 mM l-ascorbic acid. The concentrations were 1000, 500, 250, 125, 62.5, and 31.25 μM, as well as a blank (Tris buffer). Once the ammonium acetate, neocuproine, and copper (II) chloride dihydrate were added to the samples and standards, the test tubes were incubated at room temperature for thirty minutes. At this point, they were analyzed in a Hitachi U-2001 Spectrophotometer set to read at 450 nm.

For the carbohydrate analysis, the samples and standards were also prepared in duplicate by adding 20 μL of the samples or standards to each pair of test tubes containing 1 mL anthrone solution, which was prepared daily at a ratio of 1 mg of anthrone per 1 mL of 2.3:1 sulfuric acid in water. The standard curve was created using six glucose concentrations diluted in water with concentrations of 5, 2.5, 1.25, 0.6, 0.3, and 0.15 mg glucose per milliliter with the seventh standard being a blank (water). This glucose solution was prepared daily. 20 μL of each sample or standard was mixed with 1 mL of the anthrone solution, and the test tubes were incubated in a water bath at a temperature of 90 °C for five minutes. They were then placed in cold water for five minutes and then removed from the water and allowed to warm to room temperature for five more minutes. Once stabilized to room temperature, all standards and samples were read in the Hitachi U-2001 Spectrophotometer set to read at 630 nM.

### Statistics

One way analyses of variance was used followed by the Tukey test for individual differences. A *p* < 0.05 was required for statistical significance.

## 3. Results

The carbohydrate concentrations of the grapes were: Concord = 10.3 ± 1.0 μg glucose/mL; purple = 27.2 ± 1.3 μg glucose/mL; red = 25.4 ± 2.4 μg glucose/mL; and green = 26.0 ± 2.5 μg glucose/mL. The carbohydrate concentration of the green grapes was significantly different from all other grapes; *p* < 0.05.

**Figure 1:**
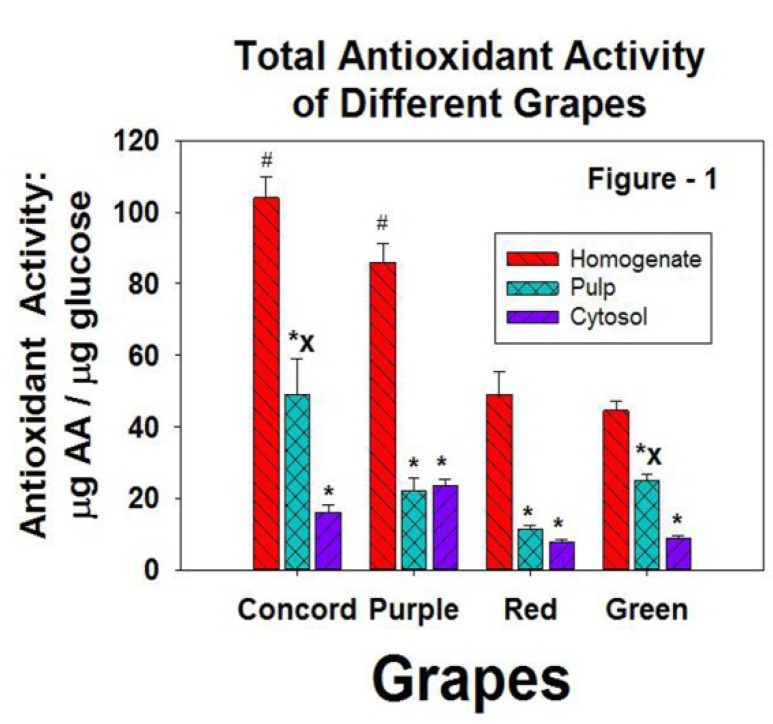
The antioxidant capacity of the homogenates (total antioxidant capacity of the grape), the pulp (a homogenate of the grape without the skin), and the cytosol (the clear supernatant after centrifugation). # = significantly higher than the antioxidant capacity of red and green grapes, *p* < 0.05. * = significantly lower antioxidant capacity of the homogenate, *p* < 0.05. *x* = significantly higher antioxidant capacity of the cytosol, *p* < 0.05.

The antioxidant capacity normalized by carbohydrate content is presented in Figure 1. The homogenate group represents the total activity of the whole grape. The concord grapes and purple grapes had significantly higher TAC in the homogenates than did the red or green grapes which interestingly had virtually the same total TAC.

The Concord grapes and green grapes had significantly higher TAC in the pulp than in the cytosol whereas the red and purple grapes had approximately the same amount.

**Figure 2:**
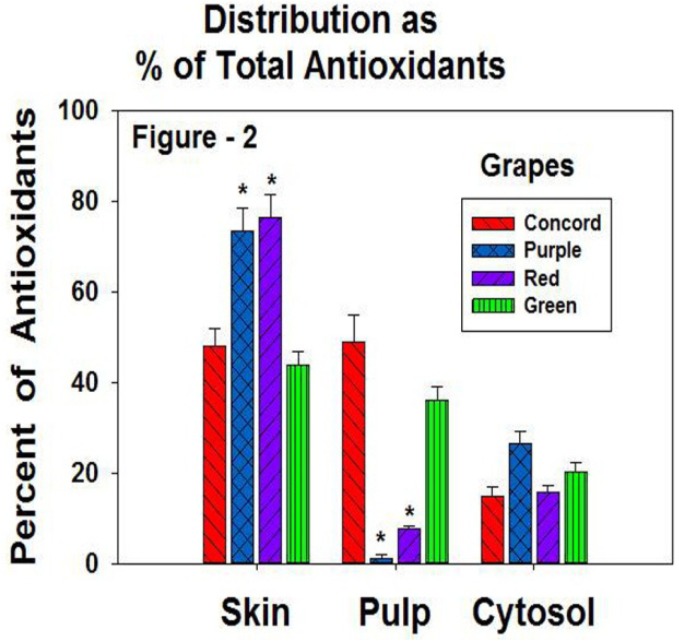
The percent of antioxidant capacity of the skin, pulp, and cytosol. * = significantly different antioxidant capacity of concord and green grapes, *p* < 0.05.

We also analyzed the data by separating the four grapes into skin, pulp, and cytosol and normalizing to percent of the total TAC of the grape (Figure 2). The red and purple grapes had a very similar percentage of antioxidant capacity in the skin, around 70%–75%, while the green and Concord grapes had approximately 45% of the capacity in the skin.

In the pulp, which included the seeds of the grapes, the concord grapes had 48% and the green grapes 38% of the total TAC. The purple and red grapes had very low antioxidant capacity in the pulp. The cytosol showed activities ranging from 15% to 26% of the total TAC.

## 4. Discussion

Our data shows that the Concord and purple grapes have a significantly higher TAC than the red or green grapes. This supports the general belief that darker grapes have more antioxidants. However, whereas the purple grapes had most of the antioxidant capacity in the skin, the Concord grapes have approximately 45% of the TAC in the skin and 45% of the TAC in the pulp. Thus, juicing these grapes and getting rid of the skin would give the Concord grapes a higher TAC than juice made from the purple grapes. It is not known whether any commercial grape juice tries to extract the antioxidants from the skin.

Interestingly, the red and green grapes did not show different antioxidant activities. Red grapes share some commonalities with purple grapes but also some others with the green. For example, the red have the majority of their polyphenols in the form of anthocyanins like the purple do while the green have mainly flavanols. When dissected, the shape, location, and size of the seeds of the red grape were virtually identical to the green grape. This contrasted with the purple grapes which had extremely inconspicuous seeds in a slightly different location.

Two hypotheses concerning the relationship between the color of the grapes and TAC are: (1) the differences in TAC are caused by either differences in the concentration of antioxidants or(2) the differences in TAC are caused by the differences in the forms of the antioxidants. It may well be that both are important. If color was a major determining factor, than it would have been expected that the red grapes would have a higher TAC than the green instead of seeing approximately the same total capacity in our results. However, similar to the differences in distribution shown between Concord grapes and purple grapes, most of the TAC of the red grapes was in the skin whereas much of the TAC of the green grapes was in the pulp. It should be noted that anthocyanins (purple and red grapes) behave differently *in vivo* than flavanols (green grapes), and studies have demonstrated that anthocyanins are strongly detected using the CUPRAC test, thus the ratio of TAC for purple grapes may be somewhat skewed [26,27,28]. Regardless, the identity of the antioxidants of each grape is still of significant importance [29,30,31,32].

The percentage of antioxidant capacity in the cytosol showed few noticeable differences between the four grapes. This suggests that the antioxidants in the four types of grapes tend to solubilize into the cytosol in the same proportions.

The TAC of grapes in general has been effectively demonstrated in studies where a grape powder suspension has been fed orally to rabbits subjected to a variety of forms of *in vivo* and *in vitro* ischemia/reperfusion and partial outlet obstruction with all models producing significant oxidative damage to the urinary bladder. These studies clearly demonstrated that oral grape ingestion reduced both oxidative damage to proteins and lipids and significantly protected the rabbit urinary bladder from both structural and contractile damage [33,34,35,36]. It should be noted that this grape suspension was standardized by the California Table Grape Commission and consisted of a freeze-dried powder of a variety of table grapes grown in California. The amount given to the rabbits would be approximately one handful of grapes per day taken by men or women.

When comparing the carbohydrates among the grapes we found that the purple, green, and red grapes have essentially the same concentration of carbohydrates whereas the Concord grapes had approximately half the carbohydrates. Since the TAC was normalized to carbohydrates, the Concord grapes would have less antioxidants per weight of grapes than the purple grapes.

## 5. Conclusions

Concord and purple grapes have significantly higher total antioxidant activities than red or green grapes which were very similar. The antioxidants in the purple and red grapes were found primarily in the skin, whereas, in the Concord and green grapes, the TAC was equally divided between skin and pulp. This study suggests that the dark color Concord and purple grapes are more beneficial in terms of antioxidants. Further studies are needed to confirm whether this is also true *in vivo*.
